# Magnetic fabric from Red clay sediments in the Chinese Loess Plateau

**DOI:** 10.1038/srep09706

**Published:** 2015-04-27

**Authors:** Hujun Gong, Rui Zhang, Leping Yue, Yunxiang Zhang, Jianxing Li

**Affiliations:** 1 Institute of Cenozoic Geology and Environment, State Key Laboratory of Continental Dynamics, Department of Geology, Northwest University, X'ian 710069, China; 2 State Key laboratory of Loess and Quaternary Geology, Institute of Earth Environment, Chinese Academy of Sciences, X'ian 710075, China; 3 Xi'an Center of Geological Survey, China Geological Survey, Xi'an 710054, China

## Abstract

Well-distributed eolian red clay in a wide area of northern China is believed to imply the onset of an ancient East Asian monsoon system since Late Miocene. Two continuous red clay sequences spanning the time interval 7–2.6 Ma and 11–2.6 Ma in the Chinese Loess Plateau was investigated to determine the magnetic orientation and grain alignment in the primary fabric of eolian sediments. The north-westerly orientation of the AMS of the eolian red clay sequences parallels the material transportation direction, which differs from the model that suggests that airborne dust from overlying loess-paleosol sequences were transported by the East Asian winter monsoon and fixed by the East Asian summer monsoon. Our results further reveal that present-day climate and air circulation patterns differ from those of the pre-Quaternary, and may provide evidence of a prevailing wind during deposition of the red clay.

The eolian loess-paleosol sequences and underlying red clay sediments ranging in age from Miocene to Holocene have been widely accepted as a unique geological archive for understanding the history and variability of the East Asian palaeomonsoon climate^1^,^2^,^3^,^4^,^5^,^6^,^7^,^8^,^9^,^10^. The mechanism driving the East Asian monsoon, particularly for the underlying Pliocene red clay formation, remains unresolved. Although many models have been developed using grain size and magnetic susceptibility to reconstruct paleomonsoon variation^5^,^7^,^11^,^12^,^13^,^14^, no direct evidence has been available—for example, indication of paleowind direction—to identify which factor played the major role in the formation of the red clay.

Heller et al.^15^ firstly measured AMS in loess and considered that loess had a uniform magnetic fabric in the Luochuan profile in China. Liu et al.^16^ demonstrated that AMS parameters could be used to evaluate the water re-working of wind-blown sediments. Thistlewood and Sun^17^ were the first to demonstrate that the anisotropy of magnetic susceptibility (AMS) was useful for determining the paleowind direction in loess-paleosol sequences. The technique was subsequently used in several studies to determine local wind deviations and paleomonsoon routes in loess^18^,^19^,^20^,^21^,^22^,^23^,^24^,^25^,^26^,^27^. Spassov et al.^28^, Deng et al.^29^, Liu et al.^30^ and Nie et al.^31^,^32^ showed in various studies that wind-borne ferromagnetic minerals account for a large proportion of the magnetic susceptibility (MS) signal in loess-paleosol and red clay sequences. Although pedogenesis and neoformed chemical alteration in the eolian particles can produce enhanced MS values, their chaotic orientation cancels out the effect^18^ and detrital contribution of particles arranged by the paleowind dominates the total AMS signal^26^. AMS measurements have also been used to identify the direction restricted by a local terrace or other disturbance^23^,^25^. By taking into account the alternating loess-paleosol sequences that clearly represent broad cyclical climate oscillations, it was found that the red clay formation accumulated in a relatively warm and stable environment^3^,^4^,^5^, with small-amplitude climate fluctuations at the end of the Miocene and Pliocene being inferred from the many intercalated carbonate nodule horizons. The geographical distribution and source areas of the red clay are also similar to those of the overlying loess. Vandenberghe et al.^11^ suggested that the north(west) winter monsoon still dominate the source of dust and the wind circulation system during the Neogene. They argued only in a small part of dust(clay and very fine silt), transporting wind was driven by the westerly. The latest studies of binary source of the loess and paleosol on the Chinese Loess Plateau (CLP) revealed by Nd-Sr isotopic and U-Pb ages of Zircon^33^,^34^,^35^,^36^ suggests that both NTP and Gobi Altay Mountains (GAMs) contribute to the dust transported to CLP. It seems from those results, the binary source of eolian dust on CLP or North China craton shift between the Qiliang Mountains (QLMs) and GAMs keeps all the way back from early Miocene to present day. However, by the zircon U-Pb ages Nie et al.^37^ reveal that the multiple individual resources of Red clay sequences were transported mostly by westerly which is different from dust material of loess sequence were transported by northwest winter monsoon.

In the present study we investigated two continuous red clay sequences from Lingtai and Shilou in the Chinese Loess Plateau to evaluate its magnetic properties and grain alignment and describe the primary eolian and water-lain sediment fabric. We were trying to further examine if water-lain deposits (redeposited loess/red clay) have a higher degree of anisotropy than that of windblown deposits since it was firstly mentioned by Liu et al.^16^ and to find if the red clay can provide evidence of a prevailing wind during deposition similar to those of the overlying loess. The samples spanned the period from 7.0 to 2.6 Ma in Lingtai and 11–2.6 Ma in Shilou.

**Sampling and experiments.** Lingtai (35°04′N, 107°39′E) is located in the central Chinese Loess Plateau, and is about 150 km north-west of Xi'an (Fig. 1). It has a semi-humid climate, with 650 mm mean annual precipitation and an average annual temperature of 8.8°C. The stratigraphy of the eolian red clay profile has been previously described by Ding et al.^2^,^4^ and Sun et al.^38^ A total of 431 oriented samples were taken at 30 cm vertical intervals from the 128 m-thick red clay for AMS measurements. Two horizons containing fossilized mammal bones were found nearby in a horizontally bedded fluvial terrace in the upper part of the red clay. The taphonomy of the fossils showed evidence of water transportation and sorting^39^,^40^ in the lower fossiliferous horizon, whereas the upper horizon was disturbed. About 77 oriented samples were taken from the fossiliferous horizons to verify their transport by water flow. Another 11-Myr eolian red clay profile was reported by Xu et al.^41^ for the Shilou profile in Shanxi Province, eastern Loess Plateau. (Figure 1). It also has a semi-humid climate, with 550 mm mean annual precipitation and an average annual temperature of 9.2°C. A total of 486 oriented samples were then taken at 15–20 cm vertical intervals from the 70 m-thick red clay. The AMS of each sample was measured by a KappaBridge KLY-4S magnetic susceptibility meter coupled with automated sample handling system, and AniSoft software using the statistical method of Constable and Tauxe^42^.

## Results

**Figure 2:**
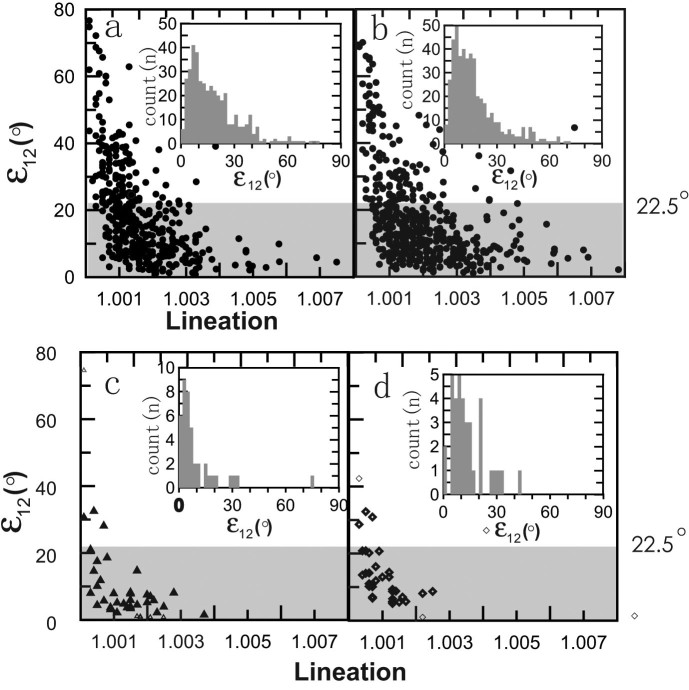
Plots of ε_12_ (the 95% confidence ellipse of K_max_ in the plane joining K_max_ and K_int_) vs lineation in the studied profiles. (a), eolian Lingtai red clay profile; (b), eolian Shilou red clay profile; (c), upper water-lain fossiliferous horizons in Lingtai; (d), lower water-lain fossiliferous horizons in Lingtai. The plots demonstrate an inverse relationship between ε_12_ and lineation.

The magnetostratigraphy of the red clay sequences were reported by Ding et al.^2^ and Xu et al.^41^ The AMS ellipsoid orientations are defined by the maximum (K_max_), intermediate (K_int_) and minimum (K_min_) principal susceptibilities. Parameters ε_12_, ε_23_ and ε_13_ are the confidence levels of the angles at the 95% probability level in determining the orientations of the principal susceptibilities (e.g., ε_12_ is the half-angle uncertainty of K_max_ and/or K_int_ in the plane of K_max_ and K_int_, where 1, 2, 3 represent K_max_, K_int_ and K_min_). An inverse relationship between ε_12_ and the magnetic lineation parameter L was observed for all the data from the wind-blown and water-lain sediments in Figure 2 due primarily to the increasing significance of random measurement errors in K_max_ for weakly lineated planes. Most samples satisfied the statistically significant level of ε_12_ < 22.5° (70.0% for eolian Lingtai sediments, 78.6% for eolian Shilou sediments, 90.2% for the upper fossiliferous horizon, and 86.1% for the lower fossiliferous horizon). Supplementary Table 1 lists the average lineation (L), foliation (F), degree of anisotropy (P), and declination/inclination of K_max_ (D-K_max_, I-K_max_) and K_min_ (D-K_min_, I-K_min_) for the eolian profile and the two water-lain fossiliferous horizons. Most samples from all studied localities exhibited the oblate magnetic fabric typical of eolian and water-lain deposition^18^,^42^,^43^.

**Figure 3:**
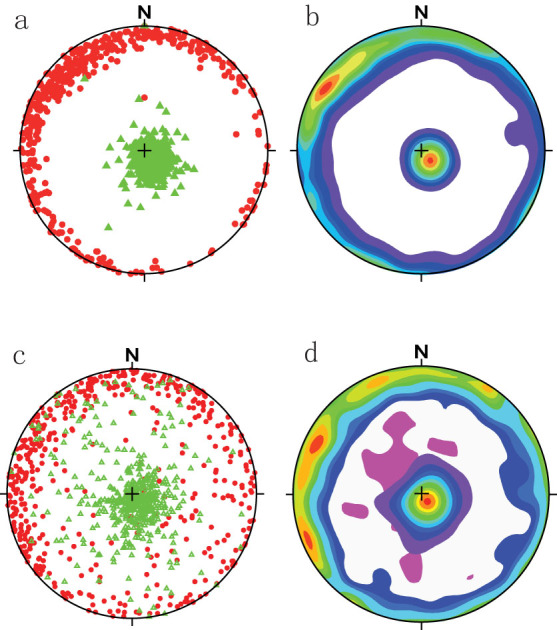
AMS results for the eolian red clay at Lingtai (a,b) and Shilou (c,d) profiles. Stereographic projection (from the lower Hemisphere) of K_max_ (red dots) and K_min_ (green triangles) (a and c); contours of K_max_ and K_min_ (b and d).

**Figure 4:**
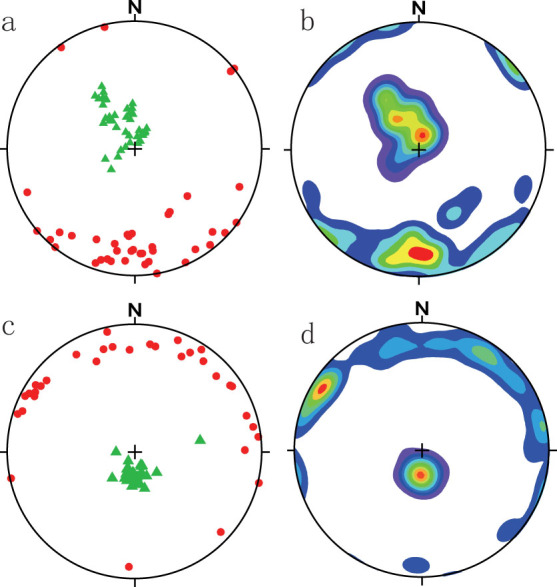
AMS results for the water-lain red clay. Red clay water-lain horizons 1 (a,b) and water-lain horizons 2 (c,d) at Lingtai: stereographic projection of K_max_ (red dots) and K_min_ (green triangles) (a and c); contours of K_max_ and K_min_ (b and d).

Figure 3 and figure 4 shows the principal orientations of the maximum and minimum susceptibility axes. The eolian red clay (Fig. 3) and water-lain horizons (Fig. 4) displayed an AMS fabric characteristic of primary deposits. The K_min_ axis was normal to the horizontal plane containing K_max_ and K_int_ for the eolian sediments (dip ~ 82°). By comparison, the foliation planes of the water-lain horizons (middle and lower part of Fig. 3) dip to the north and south at approximately 72° and 76°.

The major orientations of K_max_ and K_min_ are shown as isopleths in Figure 3. The mean directions were calculated using the “bootstrap” statistical methodology of Constable & Tauxe^42^; see (Supplementary Table 1.) A major NW magnetic lineation was observed in the eolian red clay squences (Fig. 3); the primary orientation of K_max_ was north–south in both fossiliferous horizons (Fig. 4).

## Discussion

The AMS parameters in Supplementary Table 1 imply that the sedimentary particles, including magnetite, settled and were fixed during the wet season, resulting in only minor variation in orientation between the wind-blown and water-lain sediments (including water transportation action and sorting in the fossiliferous horizons). The main difference was observed in the dip of particles (especially inclination of K_min_): I-K_max_ = 9° and I-K_min_ = 82° for the whole eolian profile(Lingtai); I-K_max_ = 13° and I-K_min_ = 72° for the upper fossiliferous horizon, and I-K_max_ = 11° and I-K_min_ = 76° for the lower fossiliferous horizon. At the same time the dip of K_max_ displays the noticeable difference between (younger) loess and (older) red clay: I- K_max_ = 3 ~ 4° from the very top layer of loess (Xifeng and Baicaoyuan profiles)^25^, I- K_max_ = 9 ~ 10° from the eolian red clay (Lingtai and Shilou), and I- K_max_ = 11 ~ 13° from reworked water-lain eolian sediments (in Lingtai). These observations might indicate the press force only gives a strong influence on the dip of K_max_ while the water current may rework not only K_max_ but also K_min_. We consider that both K_max_ and K_min_ orientations could be correlated to the predominant force (eolian or reworked water current), instead of the degree of Lineation (L), Foliation (F) and anisotropy (P) which once applied by Liu et al.^16^ in two decades before.

The tensor means (Supplementary Table 1) and isopleth contour diagram of equal K_max_ and K_min_ values (Fig. 3 and Fig. 4) are both included here. Contours are visually very descriptive but, while either approach may be used to determine paleowind direction, the contour maximum may not accurately represent the mean value for the whole particle population if the technique is used for a relatively small number of samples; for example, for the 36 specimens from the lower fossiliferous horizon, Figure 4 shows a north-westerly orientation for K_max_, whereas the tensor mean value of K_max_ and K_min_ indicates a northerly direction, consistent with the statistical analysis of the elongate bone orientations (which will be discussed further in another paper) and the terrain.

The orientation of the AMS of the water-lain horizons showed the same trend as the sorted and aligned elongate bones^39^,^40^. Although loess is eolian in origin, the reworked water-lain eolian deposits were always subject to slope-water transportation at both macro- and micro-scales while the eolian red clay as the same as the loess has no such relations. And there were only difference of dip of Kmax (Incliation) existed between the eolian loess and red clay.

In previous studies, spatial analysis of grain size distributions in the red clay^3^,^4^,^5^ showed that grain sizes in the red clay do not decrease significantly from north to south, which is in sharp contrast to the particle size distribution of the overlying loess–paleosol sequence where a marked southward decrease in particle size was observed. They concluded for the first time that significantly different wind systems had been responsible for transporting the red clay and the Pleistocene loess: the loess was transported by northerly winter monsoonal winds, and the red clay materials were transported mainly by winds from the west. However, Miao et al.^12^ argued that the grain size of the Red Clay indicates the Red Clay transported mainly by a weaker winter monsoon (northerly low-level winds) instead of westerly. Vandenberghe et al.^11^ inferred that the Red Clay is mainly composed of two components: (1) the finest grain-size fraction of silt and clay in the Red Clay was transported by the westerly (only a minor part of the clay is of pedogenic origin); (2) a coarser silt fraction that is very similar to the silt fraction in the upper loess sequence was supplied over relatively short distances by near-surface, northwesterly winds—the winter monsoon played a major role in the air circulation during the Neogene.

The weaker monsoon system during the Tertiary was due to the lower altitude and thus less prominent impact on the circulation pattern, of the Tibetan Plateau. The controversial issue about Red Clay source continues for lack of detailed provenance work undertaken on the Red Clay sequences that can trace the source region and/or determine the paleowind direction. The latest studies by tracing the source of loess and Red Clay on the CLP using the U-Pb ages of zircon and Nd-Sr isotopic composition may help review of the various desert areas to show a range in the provenance of eolian sediments and shed light on the understanding of causes of long term Asian aridification. For examples, binary source of the loess and paleosol on the CLP revealed by Nd-Sr isotopic and U-Pb ages of Zircon^33^,^34^,^35^ suggests that both NTP and Gobi Altay Mountains (GAMs) contribute to the dust transported to CLP. NTP dust can be transported by westerly wind, Yellow River, and northwesterly winds (winter monsoon) from the Alxa arid land (AALs)). It is suggested that the arid lands between Qilian Mountain and GAMs in AALs are the main resource regions for deposits in the CLP. Silicate Nd and Sr isotopic signatures and zircon U-Pb ages from Red Clay sequence, however, have observed several stages source changes or air circulation models^36^,^37^. Schematic diagram of dust transport routers by Nie et al.^37^ indicated that the relative importance of monsoon versus westerly wind. They concluded that the provenance of late Miocene lowermost Red Clay (8–5.5Ma) is either sourced from the Qaidam Basin via the lower-level westerly, or transported by the fluvial system from the Liupan Mountains. The middle (5.5–4) and upper parts (4–2.6) of Red Clay are sourced mainly from Taklamakan desert and mixed areas via the westerly too. Only the Quaternary loess-paleosol sequences is may be sourced from the proximal deserts via the northwest winter monsoon, which in turn may be the materials from the mountains like GAMs and Qilian mountain, transported by the fluvial systems. Nevertheless, Nd and Sr isotopic records of the eolian sources from the Miocene appeared some different from their studies, for example, Chen et al.^36^ observed the dust source shift is governed by the competition of detrital contribution from QLMs and GAMs in three stages of source change over the past 22Ma. From 22 Ma to 7 Ma, material contributions from Qilian Mountains increase rapidly. During 7–1.2 Ma, relative constant Nd and Sr isotopic compositions of the eolian deposits suggested the ration of detrital contribution between QLMs and GAMs kept relatively constant. Since 1.2 Ma, the detrital contribution of GAMs rises rapidly. It seems from their results, the binary source of eolian dust on CLP or North China craton shift between the QLMs and GAMs keeps all the way back from early Miocene to present day which imply a monsoon air circulation since the early Miocene.

Recent studies have pointed out that the AMS ellipsoloid orientation parallels the imbrication of the grains, evidence for a prevailing summer monsoon for the loess-paleosol sequence^26^. The northerly winter monsoonal wind carried the eolian materials to the area, then the particles, including magnetite, were rearranged, settled and fixed during the wet, windy summer season. Ge et al.^27^ reported the results of AMS measurements of loess at sites exhibiting varying slope angles in the CLP. Their results show that within the same region, magnetic lineations are clustered along similar orientatons despite differences in slope exposure this may help confirm the ability of the AMS to record the paelowind direction. They also concluded that the AMS of Chinese loess is mainly determined by the regional surface wind flow that occurred during course of dust accumulation, rather than by the large-scale atmospheric circulation. In this paper, we compared the AMS of the wind-blown and water-lain sediments which also provide supports for that the AMS of the eolian sediments may faithfully reflect the paleowind directions. The present study of the orientation of the magnetic fabric of the underlying red clay sequences reveals that the highest susceptibility materials are grouped in the NW quadrant and the lowest susceptibilities are clustered in the SE quadrant, evidence that north-westerly winds played a major role in the orientation of the AMS ellipsoid before the Pleistocene. In comparison, the northwest orientation of magnetic grains for red clay probably indicates that the summer monsoon was not as strong as the Quaternary to significantly affect orientation or red clay. Or more likely, the finer grain size and more tightly-packed grains (less pore space) of red clay in contrast to loess make red clay easy to retain orientation of deposition and less sensitive to reorientation associated with precipitation.

Comparing the previous debates about either winter monsoon or westerly dominate the transport source and air circulation, we sill have a limitation in this study. We could only find in our result there is a great change about the paleowind directions from southeast to northwest. As the well-known chaotic influence on the results of AMS, we could not get a better-detailed result than the statistic method. For example, we could not get the tempo variations in a vertical scale at present with less samples at each stage, instead, we could only put the all samples together to take a statistic analysis. East Asian summer monsoon increased and had a much stronger intensity than that of winter monsoon during Quaternary^26^. While we did not find the summer monsoon had such great intensity at Neogene. It might be a reason for the fast increasing of desertification in the northwestern China, new debates might arise as the history of NTP uplift has remained uncertain^3^,^5^,^11^,^12^,^33^,^34^,^35^,^36^,^37^. Mutilple researches have shown that either Westerly or Winter monsoon dominate the air circulations during the Neogene is possible, each view point has some evidence to support them, we still do not have a better method to identify the truth of them. Much more future works from the geochemical isotopic measurements might help us better understand the scenarios. For examples, take multiple sample measurements of both U-Pb dating of zircon and Nd-Sr isotopic composition at the same sections including loess and Red Clay sequence may improve the understanding of the provenance of eolian sediments since the early Miocene. AMS measurements also need to find a new filter technique, which is like the magnetic cleaning in the paleomagnetic data to excluded the chaotic errors from pedogenesis, vegetation grown, compaction and eluviation.

## Conclusions

The study describes the AMS measurement and analysis for the eolian red clay in central and eastern Loess Plateau. The orientations of the maximum and minimum AMS axes of the sedimentary particles in both the eolian and the water-lain sequences were determined with a view to revealing the paleowind and paleocurrent directions.

The AMS ellipsoid orientation of Red Clay towards the northwest was determined by the material transportation direction. It was considered that the materials of the red clay formation were transported and fixed by northwesterly winds. Although the chaotic results of AMS indicate it is influenced by many factors with no better method to solve this problem. It seems statistic methods of AMS based on large samples could shed light on the understanding of AMS analysis. And in this study (see Supplementary Table SM-1), water-lain red clay do not have a higher degree of anisotropy than that of windblown deposits as it was only reported by Liu et al.^16^

## Supplementary Material

Supplementary Information Supplementary Information

## Figures and Tables

**Figure 1:**
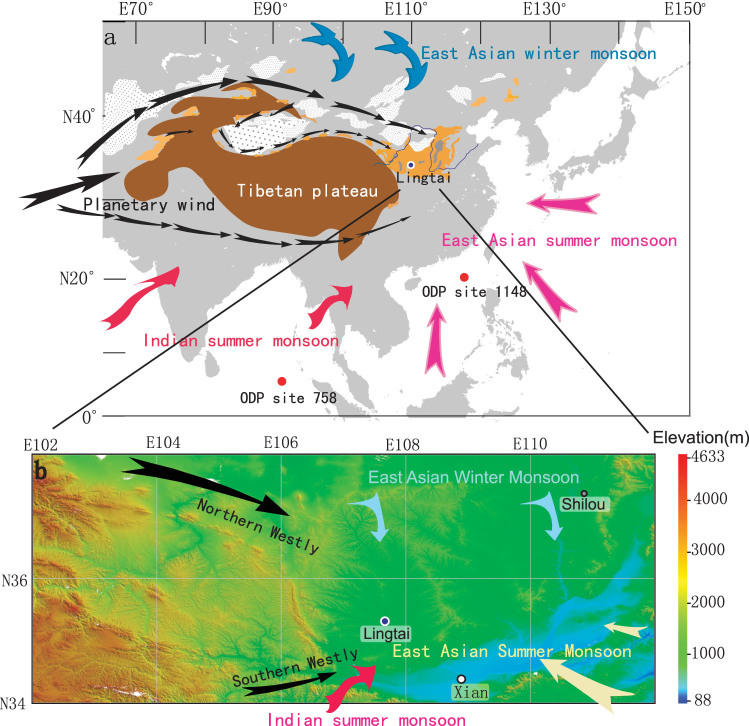
Schematic map of the Chinese Loess Plateau showing the sampling locations in China. (a), Loess Plateau and wind regime in the Asian interior with localities of paleoclimatic studies. (b), Topography of the studied sections and adjacent areas. (The map has been modified from its original version generated by the Global Mapper software).
