# Supplementary material for: Magnetic fabric from Red clay sediments in the Chinese Loess Plateau
Source: Sci Rep. 2015 Apr 27;5:9706. doi: 10.1038/srep09706 (PMC5386194; doi:10.1038/srep09706)
Supplement: Supplementary Information [file srep09706-s1.doc]

**SUPPLEMENTARY MATERIALS**

**Magnetic fabric from Red clay sediments in the Chinese Loess Plateau**

Hujun Gong^a,b^, Rui Zhang^a,b,c*^, Leping Yue^a,b,c^, Yunxiang Zhang^a,b^ and Jianxing Li^c,a^

1. Institute of Cenozoic Geology and Environment, State Key Laboratory of Continental Dynamics, Department of Geology, Northwest University, X’ian 710069, China
2. State Key laboratory of Loess and Quaternary Geology, Institute of Earth Environment, Chinese Academy of Sciences, X’ian 710075, China
3. Xi’an Center of Geological Survey, China Geological Survey, Xi’an 710054, China.

* Corresponding author: E-mail: ruizhanggeo@gmail.com; Tel: +86-29-85246140; Fax: +86-29-87316977

*Scientific Report*

*2014*

Table SM-1 Averaged parameters of the AMS

| Horizon | *n* | D-K_max_/I-K_max_ | D-K_min_/I-K_min_ | Lineation | Foliation | Degree of anisotropy |
| --- | --- | --- | --- | --- | --- | --- |
|  |  |  |  |  |  |  |
| Water-lain fossiliferous horizon 1（Lingtai） | 41 | 204°/13° | 338°/72° | 1.001 | 1.019 | 1.020 |
| Water-lain fossiliferous horizon 2（Lingtai） | 37 | 348°/11° | 181°/76° | 1.001 | 1.008 | 1.009 |
| Whole Eolian red-clay profile (Lingtai) | 413 | 310°/9° | 207°/82° | 1.002 | 1.012 | 1.014 |
| Eolian red-clay (Shilou profile) | 486 | 303°/10° | 236°/81° | 1.002 | 1.007 | 1.009 |
| Eolian Loess (Xifeng profile)[Zhang et al., 2010] | 521 | 128°/3° | 327°/83° | 1.001 | 1.004 | 1.006 |
| Eolian Loess (Baicaoyuan profile) [Zhang et al., 2010] | 187 | 91°/4 ° | 307°/85° | 1.002 | 1.006 | 1.007 |
